# An Integrated H-G Scheme Identifying Areas for Soil Remediation and Primary Heavy Metal Contributors: A Risk Perspective

**DOI:** 10.1038/s41598-017-00468-2

**Published:** 2017-03-23

**Authors:** Bin Zou, Xiaolu Jiang, Xiaoli Duan, Xiuge Zhao, Jing Zhang, Jingwen Tang, Guoqing Sun

**Affiliations:** 1 Key Laboratory of Metallogenic Prediction of Nonferrous Metals and Geological Environment Monitoring, Ministry of Education, Changsha, Hunan 410083 China; 2 School of Geosciences and Info-Physics, Central South University, Changsha, Hunan 410083 China; 3 School of Energy and Environmental Engineering, University of Science and Technology Beijing, Beijing, 100083 China; 4 State Key Laboratory of Environmental Criteria and Risk Assessment, Chinese Research Academy of Environmental Sciences, Beijing, 100012 China; 5 Hunan provincial communications planning, survey and design institute, Changsha, Hunan 410008 China

## Abstract

Traditional sampling for soil pollution evaluation is cost intensive and has limited representativeness. Therefore, developing methods that can accurately and rapidly identify at-risk areas and the contributing pollutants is imperative for soil remediation. In this study, we propose an innovative integrated H-G scheme combining human health risk assessment and geographical detector methods that was based on geographical information system technology and validated its feasibility in a renewable resource industrial park in mainland China. With a discrete site investigation of cadmium (Cd), arsenic (As), copper (Cu), mercury (Hg) and zinc (Zn) concentrations, the continuous surfaces of carcinogenic risk and non-carcinogenic risk caused by these heavy metals were estimated and mapped. Source apportionment analysis using geographical detector methods further revealed that these risks were primarily attributed to As, according to the power of the determinant and its associated synergic actions with other heavy metals. Concentrations of critical As and Cd, and the associated exposed *CRs* are closed to the safe thresholds after remediating the risk areas identified by the integrated H-G scheme. Therefore, the integrated H-G scheme provides an effective approach to support decision-making for regional contaminated soil remediation at fine spatial resolution with limited sampling data over a large geographical extent.

## Introduction

Soils are being increasingly polluted as a result of growing urbanization, deforestation and industrialization. The wide spread and hazards of soil pollution are detrimental for both the environment and human beings^1–3^. Among soil pollutants, heavy metals are extremely hazardous due to their non-degradability, leaching ability, and massive accumulation^4–6^. With the boost of urbanization and industrialization, China has become the world’s leading heavy metal producer, resulting in the contamination of soils with high concentrations of heavy metals. These contaminated soils pose serious threats to human health and social stability^7, 8^.

To mitigate the harmful effects of heavy metal pollution, the Chinese government has announced the National Remediation Project of Heavy Metal Contaminated Soil (NRP-HMCS) across the country. However, its effects are greatly reduced due to inadequate financial support and inadequate recognition of areas with heavy metal pollution in need of remediation^9, 10^. Soil quality standards have been employed in China since 1995 with the release of ‘Environmental Quality Standard for Soils GB 15618-995’ to assess soil pollution. However, areas with concentrations exceeding the standard do not necessarily pose a serious health risk caused by polluted soil because of spatially differentiated population distribution and exposure pathways. In other words, heavily polluted areas might not necessarily match areas with high health risk^11^. Therefore, a risk-based identification of preferential areas for soil remediation could be a more effective way to ensure the success of NRP-HMCS in China with limited financial support.

To access the risk of heavy metal contaminated soil, the US Environmental Protection Agency (USEPA) has released a human health risk assessment model that comprehensively considers heavy metal concentrations in soil and related population exposure^12, 13^. Generally, the human health risk assessment model employs carcinogenic and non-carcinogenic risk indices to measure potential population exposure risks caused by heavy metal contamination^2^. While these indices can be used as a criterion to indicate the necessity of soil remediation at a polluted site, they have not been fully implemented to spatially target heavy metal-contaminated areas at high resolution over a large geographical extent^14, 15^ automatically, with limited site-based sampling data.

Moreover, similar to widely used methods such as principle component analysis^16^, probabilistic distribution^17^ and multivariate regression^18–20^ for source apportionment analysis in environmental health field, the human health risk assessment model can only reveal the contribution of a specific heavy metal to the total heavy metal based carcinogenic and non-carcinogenic risks, based on data from discrete sampling sites. This type of source apportionment analysis is prone to non-specific results for the key heavy metals in the soil remediation process at an area unit, as it seldom considers the risk differences between sample sites, or the joint contribution of any two specific pollutants to the total risk.

Fortunately, the geographical detector (Geo-detector), a novel surface data analysis tool, was recently developed to widely measure contributions of various independent factors to the distribution of dependent patches with the power of determinant (i.e., *PD*)^14, 21, 22^. While Geo-detector has theoretically advanced site based source apportionment analyses through grid based areal surface computations, no study has been reported thus far exploring its feasibility in accurately detecting contributions of heavy metals to associated health risks over a large geographical area. Therefore, this study proposes an innovative integrated scheme, combining human health risk assessment and geographical detector methods (H-G scheme) based on geographical information system (GIS) technology. Specifically, under the integrated H-G scheme, solutions to accurately identify areas with heavy metal pollution that require soil remediation will first be developed using the human health risk assessment model and GIS spatial interpolation method. Then, the primary heavy metal contribution to related health risk will be detected using Geo-detector in a case study area. At last, the reliability analysis of the integrated H-G scheme is also conducted through comparing the concentration surfaces of critical heavy metal pollutants, as well as the associated exposed risks in the areas identified by the integrated H-G scheme before and after remediation.

## Results

### Descriptive statistics of heavy metal concentrations

Table 1 displays the descriptive statistics of Cd, As, Cu, Hg and Zn concentrations in the topsoil at 33 sampling locations before remediation. Clearly, concentrations of different heavy metals show considerable variations across the industrial park. Concentrations of Cd, As, Cu, Hg, and Zn range from 1.0 to 7.1 mg/kg, 2.5 to 40.4 mg/kg, 0.3 to 86.1 mg/kg, 0.2 to 1.5 mg/kg, and 12.9 to 454.4 mg/kg, respectively. In comparison with grade II values of soil environmental quality standard of China (MEPPRC 1995), the average concentrations of Cd and Hg in the study area are 10.0- and 1.7-times greater than the regional safety values, whereas the maximum concentrations of Cd, As, Cu, Hg and Zn are 23.7-, 1.4-, 1.7-, 5.0- and 2.3-times the national standard values, respectively.

**Table 1:** Statistics of heavy metal concentrations at 33 locations (at 0–20 cm in depth) in the study area.

Heavy metal (mg/kg)	Cd	As	Cu	Hg	Zn
Min	1.0	2.5	0.3	0.2	12.9
Max	7.1	40.4	86.1	1.5	454.4
Mean	3.1	12.3	31.4	0.5	178.7
Standard value (Grade II)^a^	0.3	30	50	0.3	200
Mean fold	10.0	—	—	1.7	—
Maximum fold	23.7	1.4	1.7	5.0	2.3

Note: Heavy metal concentrations more than the secondary criterion are referred to as “soil pollution”.

^a^Grade II of environmental quality standards values for soils of China (MEPPRC 1995).

### Spatial patterns of heavy metal concentrations

**Figure 1:**
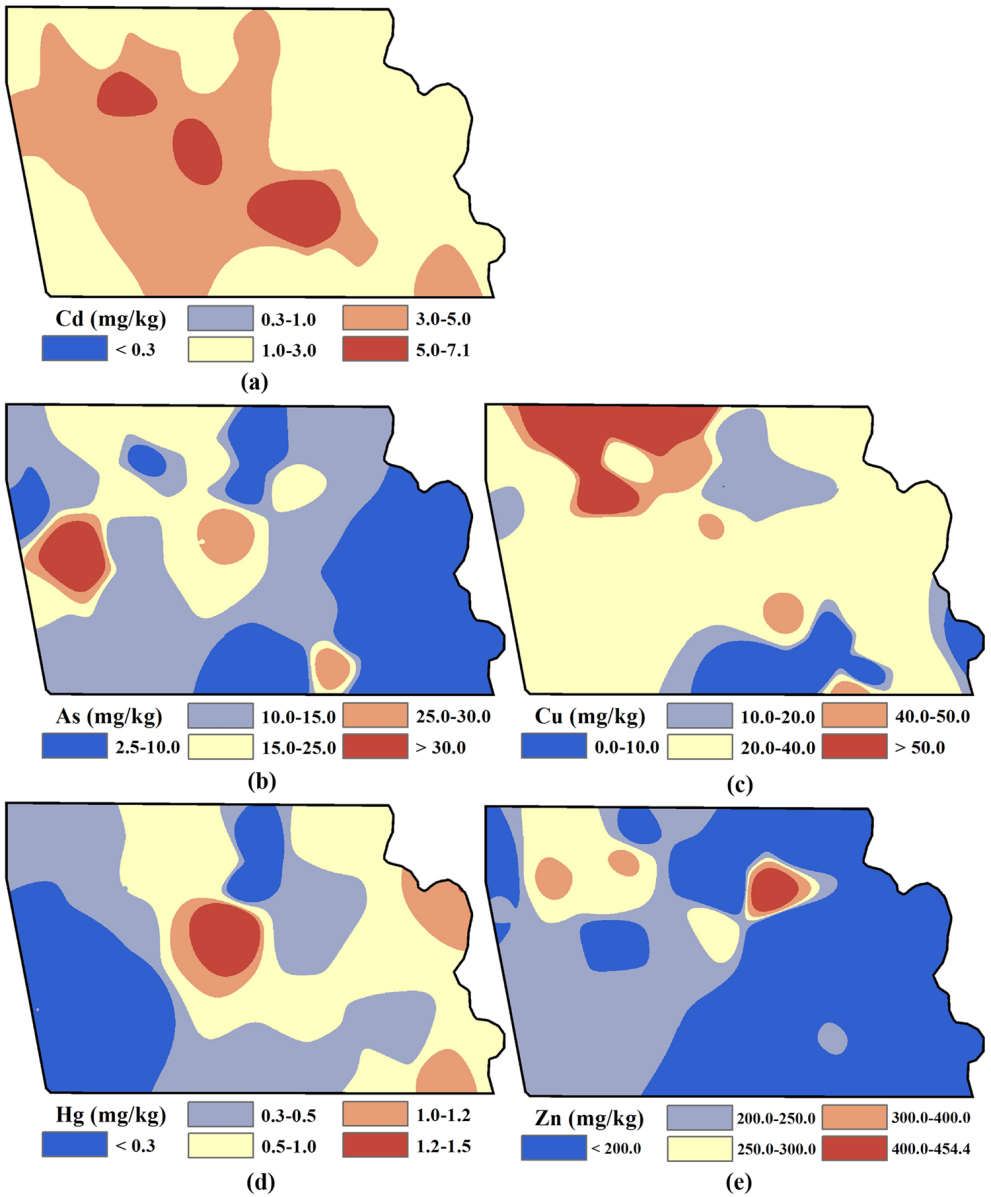
Spatial distribution of the heavy metal concentrations before remediation: (**a**) Cd; (**b**) As; (**c**) Cu; (**d**) Hg; (**e**) Zn (Note: ArcGIS 10.1 was used to create the map in this figure, http://www.esrichina.com.cn/2015/0107/2830.html).

The Inverse Distance Weighted (IDW) interpolated spatial patterns of Cd, As, Cu, Hg and Zn concentrations in the topsoil of the industrial park before remediation are presented in Fig. 1 with the accuracy listed in Table 2. Figure 1 shows that global distributions of heavy metals have elevated concentrations close to residential regions (the industrial sites). For Cd concentration, the hotspots with the highest concentration are recorded near the residential region and are up to 23.7 times the threshold value. For As, only hotspots in the west non-residential region have concentrations over the standard value (30 mg/kg). Cu was concentrated around the north residential region, accounting for approximately 1/5 of the whole industrial park. Areas with Hg concentrations greater than the standard value were mainly distributed in the northern residential region and eastern non-residential region of the industrial park. In addition to the non-residential region in the northeast, most areas have Zn concentrations slightly (i.e., 1.0–2.0 times) greater than the standard value (200 mg/kg).

**Table 2:** Cross-validation results for IDW interpolation of heavy metals.

	ME (mg/kg)	MRE (%)	RMSE (mg/kg)
As	4.11	10.46	5.00
Cu	17.17	14.99	16.12
Zn	18.43	11.93	15.39
Hg	0.20	1.36	0.09
Cd	0.98	7.46	0.52

### Human health risk caused by heavy metals

**Figure 2:**
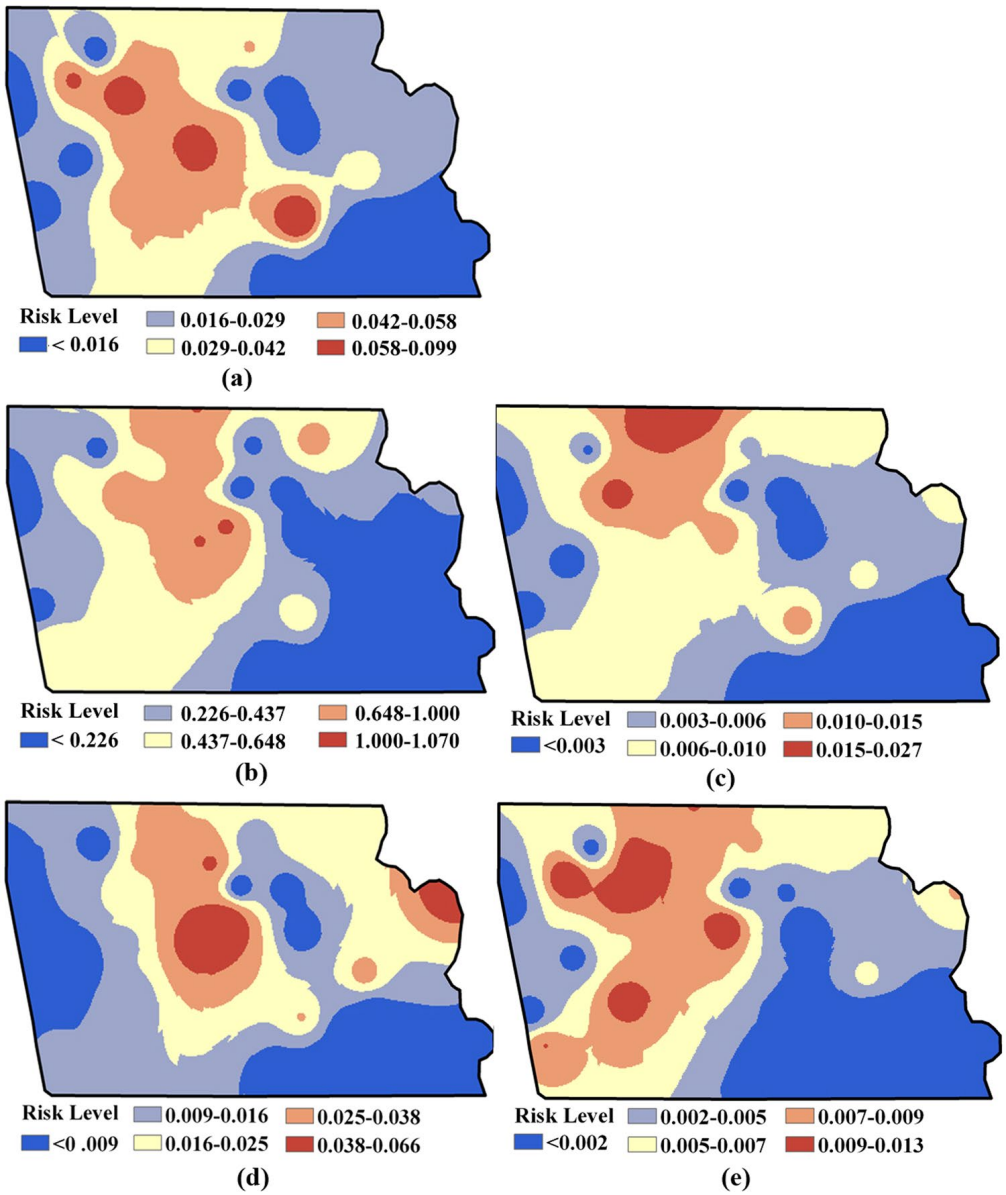
Spatial distribution of non-carcinogenic risk for each specific heavy metal before remediation: (**a**) Cd; (**b**) As; (**c**) Cu; (**d**) Hg; (**e**) Zn (Note: non-carcinogenic diseases might be caused by heavy metal exposure if *HI* > 1; ArcGIS 10.1 was used to create the map in this figure, http://www.esrichina.com.cn/2015/0107/2830.html).

**Figure 3:**
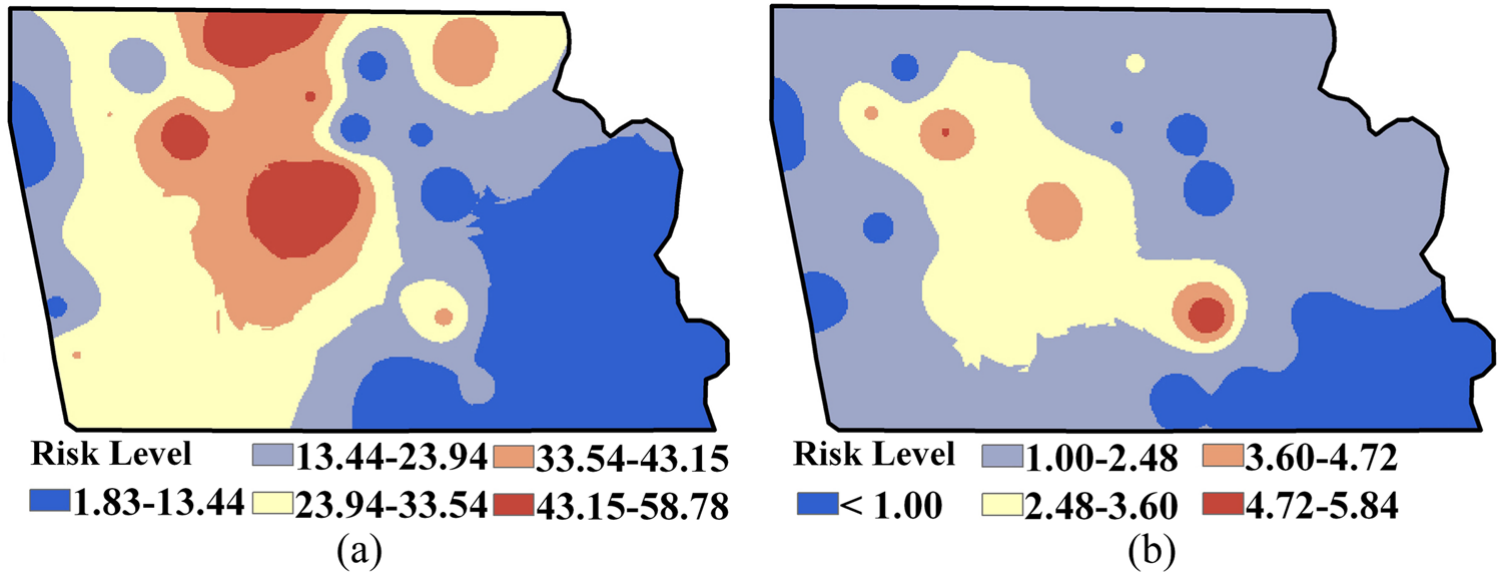
Spatial distribution of carcinogenic risk before remediation for As (**a**) and Cd (**b**) (Unit: 10^−6^) (Note: generally cancer might be caused by heavy metal exposure if *CR* > 1; ArcGIS 10.1 was used to create the map in this figure, http://www.esrichina.com.cn/2015/0107/2830.html).

Figures 2 and 3 show the non-carcinogenic risk (*HI*) and carcinogenic risk (*CR*) resulting from human exposure to heavy metal contamination in the study area before remediation. It is clear that the non-carcinogenic risks of the industrial park for Cd, Cu, Hg and Zn in soil are <1.0 (Fig. 2(a,c–e)); for As, is partly >1.0 (Fig. 2(b)). Meanwhile, Fig. 3(a) shows that the carcinogenic risks for As (varying from 1.83E-06 to 5.88E-05) across the entire industrial park are greater than the standard acceptable risk safety level for a single contaminant (1.0E-06); areas with the highest carcinogenic risk cluster in the central industrial park. For Cd contamination, the elevated carcinogenic risk areas cover almost the whole industrial park, with the highest risk recorded at 5.84E-06. However, there is still a small area with carcinogenic risk under 1.0 in the southeastern corner of the industrial park (Fig. 3(b)).

**Figure 4:**
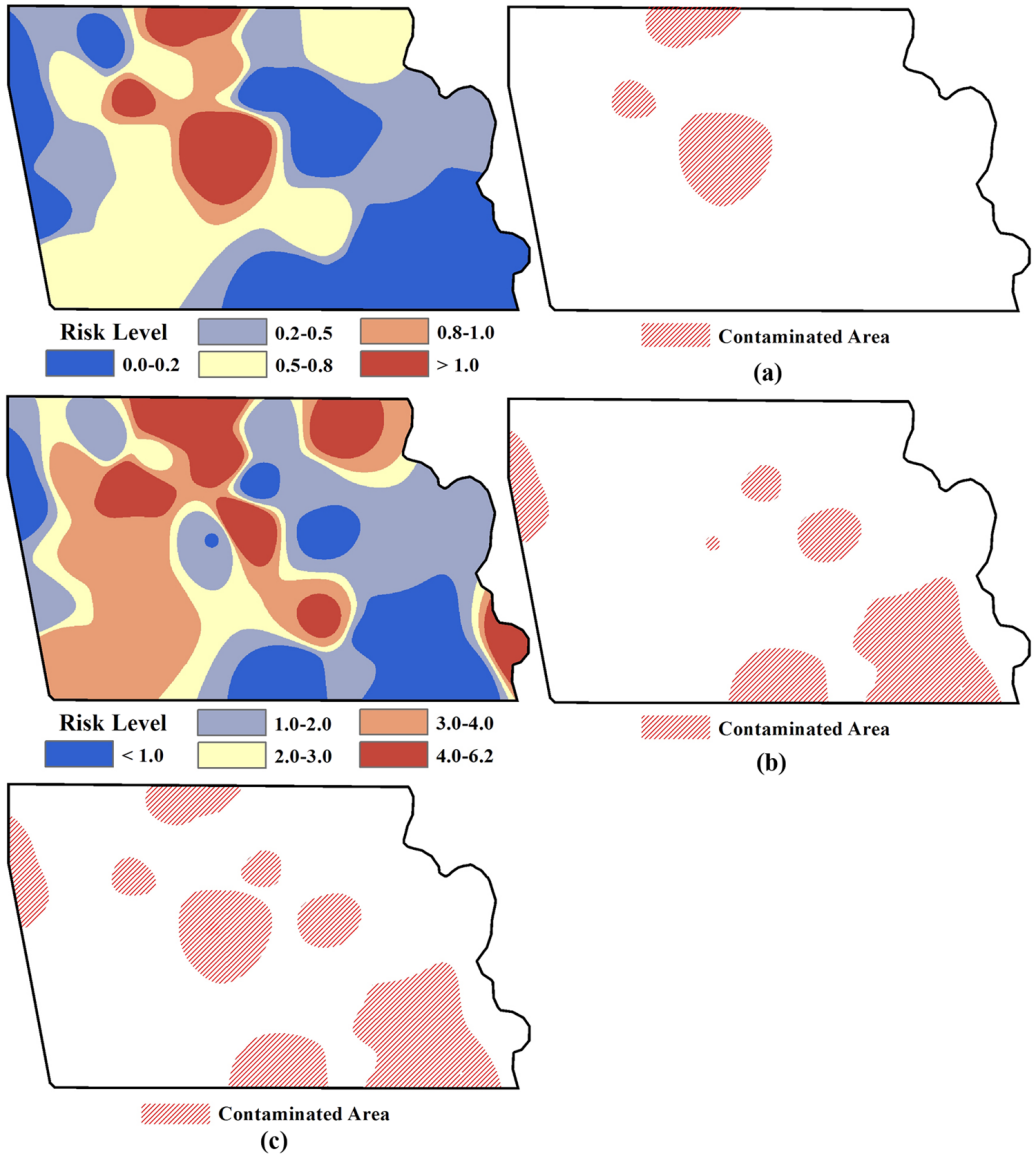
Spatial distribution of health risks and the contaminated areas identified before remediation: (**a**) *CR* risk (Unit: 10^−4^) and associated contaminated area; (**b**) *HI* risk and associated contaminated area; (**c**) the overall contaminated area (Note: ArcGIS 10.1 was used to create the map in this figure, http://www.esrichina.com.cn/2015/0107/2830.html).

Figure 4 displays the distribution maps of multiple heavy metals’ *CR* risk, *HI* risk and overall risk (i.e., overlaid raster of *CR* and *HI* risks), as well as the identified associated contaminated areas. As shown in Fig. 4(a), the contaminated areas where *HI* risks are higher than the acceptable level (i.e., 1.0) are mainly concentrated in the middle and northern industrial park, accounting for 8.1% of the entire area. Meanwhile, areas with *CR* risks over the acceptable level (i.e., 1.0E-04) are located in the south-eastern and partly in the western industrial park, with a proportion up to 18.2% (Fig. 4(b)). In addition, the results combined in Fig. 4(c) also indicate that the contaminated areas identified as overall risk areas are concentrated sparsely in the middle residential region and the non-residential region in the northeast, accounting for approximately 26.0% (partly superposed, located in the middle residential region) of the industrial park.

### Contributions of heavy metals to human health risk

Table 3 presents the contribution of heavy metal concentration in topsoil to health risk before remediation. The influences of single factors on health risk, listed in the order of *PD* values are: As (0.460) > Cu (0.312) > Zn (0.305) > Cd (0.267) > Hg (0.158). As concentration plays the greatest role in overall risk, followed by Cu, Zn and Cd. Hg contributes slightly to the overall risk. Meanwhile, the joint impacts of two factors reveal the interactive effects between As and Cd (0.682), As and Cu (0.654), As and Hg (0.795), As and Zn (0.620), Cd and Cu (0.600), Cd and Hg (0.547), Cd and Zn (0.611), Cu and Hg (0.679), Cu and Zn (0.593), Hg and Zn (0.697) appear to be stronger than the impacts of the corresponding separate factors. Even those factors with lesser interaction impacts are likely to enhance their separate effects on human health. However, after interacting Cd with Hg, and Zn, Cu, and Hg with As, as well as Cu with Zn, the relationships between them are bi-linear.

**Table 3:** Single factor and joint factors’ detection by Geo-detector.

Determinants		Cd	Hg	Zn	Cu	As
PD for single factor		0.267	0.158	0.305	0.312	0.460
PD for joint factors	Cd					
	Hg	0.547				
	Zn	0.611	0.697			
	Cu	0.600	0.679	0.593		
	As	0.682	0.795	0.620	0.654	

### Reliability analysis of integrated H-G scheme

**Figure 5:**
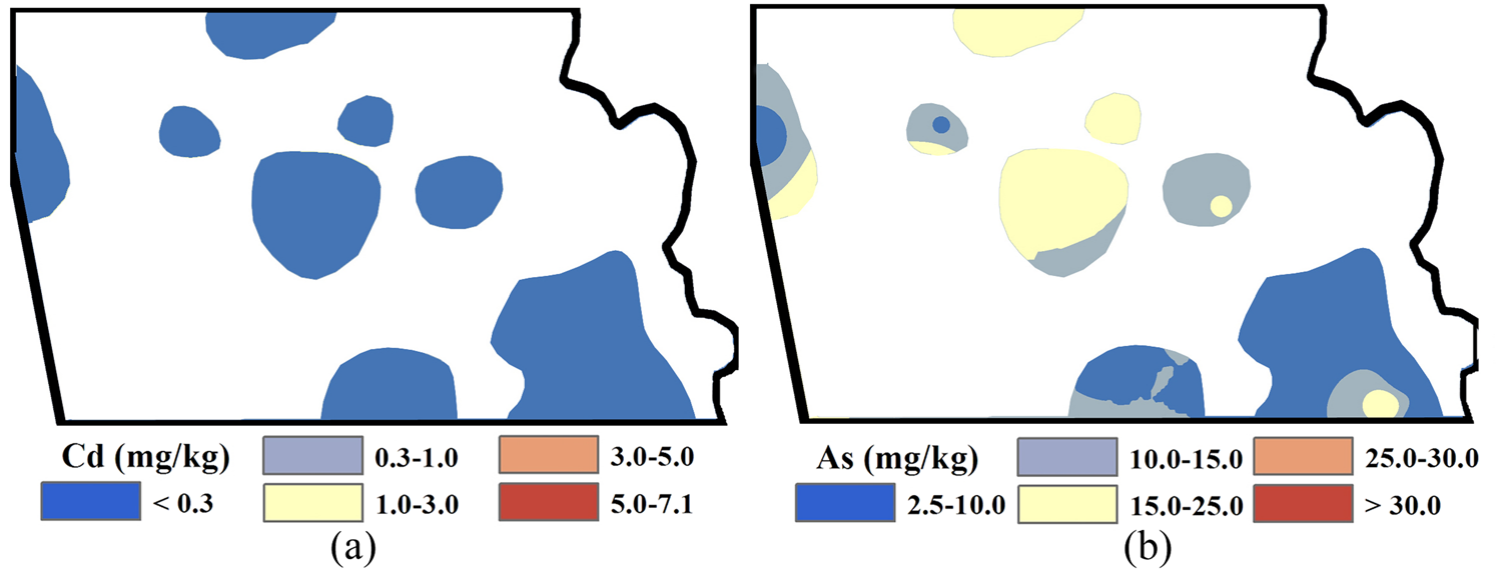
Spatial distribution of the heavy metal concentrations for Cd (**a**) and As (**b**) after remediation (Note: ArcGIS 10.1 was used to create the map in this figure, http://www.esrichina.com.cn/2015/0107/2830.html).

**Figure 6:**
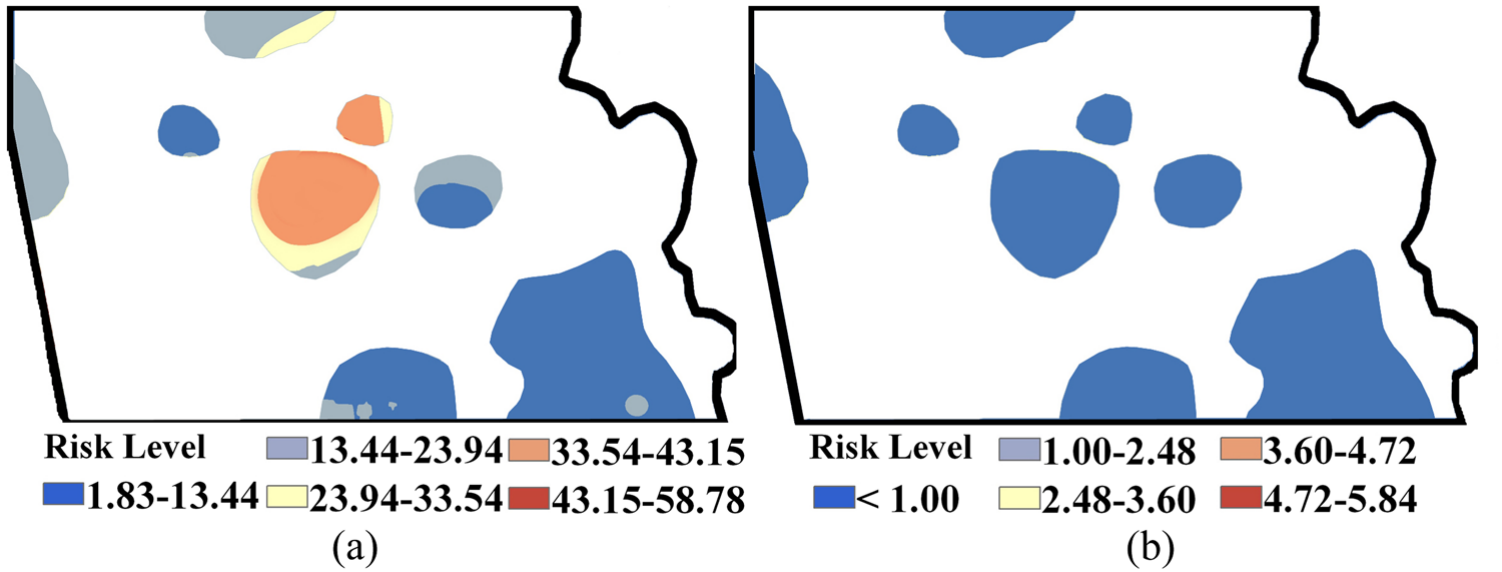
Spatial distribution of carcinogenic risk for As (**a**) and Cd (**b**) after remediation in areas identified by the H-G scheme (Unit: 10^−6^) (Note: ArcGIS 10.1 was used to create the map in this figure, http://www.esrichina.com.cn/2015/0107/2830.html).

Figures 5 and 6 show the concentration surfaces of As and Cd, as well as the associated exposed *CRs* selected to assess the reliability of the integrated H-G scheme in identifying the heavy metal polluted soils for remediation in study area. Comparing with the results in Fig. 1, it is clear that the relative high concentrations of As and Cd in the area necessary for remediation identified by the integrated H-G scheme are obviously cut down and are close to the corresponding grade II thresholds of soil environmental quality standard of China after remediation. Comparison of Figs 3 and 6 also reveals that the associated *CRs* in the area are significantly reduced accordingly, and are finally under the acceptable risk thresholds in this area considering the background concentrations of heavy metals.

## Discussion

This study analyzed the heavy metal contamination of topsoil based on data collected from a renewable resource industrial park in mainland China. In this process, the human health risk assessment model was applied to judge soil contamination risk and identify contaminated areas that require remediation. The primary pollutant of the total heavy metal contamination was detected using the Geo-detector method. Consequently the reliability of the integrated H-G scheme in identifying the heavy metal polluted soils for remediation was assessed through comparing the concentration surfaces of critical heavy metal pollutants, as well as the associated exposed *CRs* in the identified areas before and after remediation. The results highlight that the innovative integrated H-G scheme combining human health risk assessment and the Geo-detector methods based on GIS mapping technology is helpful for identifying areas for soil remediation and the primary heavy metal contributors with limited site samples. Meanwhile, compared to the traditional cost intensive and limited representation point sampling strategy, the integrated H-G scheme demonstrates a cost advantage. Using the IDW interpolation method provides a fine resolution soil remediation investigation through a continuously interpolated surface of health risks based on a limited number of site sampling inputs.

The descriptive statistical evidence confirmed high heterogeneity and variability of the heavy metal concentrations in the sampled sites over the industrial park, which might result from current or past anthropogenic sources^12, 23^. As a renewable resource industrial park, the sources of heavy metals in soil are mainly from disassembly of used electronic devices, oil refining from scrap automobile tires, and polluted surface runoff^15^.

Research providing a health risk assessment of heavy metals in Chinese soils dates back to the 1980s^24^. Previous health risk results were used for site-based qualitative assessment of heavy metal contamination, but the H-G scheme proposed in this study could theoretically amend the currently used strategy by mapping distributions of heavy metal concentrations and risk areas with the aid of GIS interpolation and spatial analysis technologies. As a result, the fine-scale distribution of areas with heavy metal pollution that require remediation could be more accurately identified^25^. The obvious differences between these areas identified based on risk and those areas simply recognized using soil quality standard concentrations confirm the importance of the H-G scheme. The H-G scheme can shape an assessment indicator by comprehensively considering soil pollution concentration and human exposure parameters and consequently determining the necessity of soil remediation rather than directly selecting contaminated areas based on pollutant concentration. And this in fact has been confirmed in the further reliability analysis of the integrated H-G scheme in identifying the areas with ‘critical’ heavy metals’ pollution that require soil remediation in this study. This would enable the H-G scheme to be widely employed to target real risk areas that require soil remediation in China’s national soil remediation project and consequently save financial resources nationwide.

In soil, the combined toxicity of multiple heavy metals might pose a higher potential risk to organisms and ecosystem health than that of a single heavy metal. In addition, with sewage irrigation, chemical fertilizers and sludge, compound pollution poses a significant threat^26^. One reason for the interactive contamination in this study might be that the industrial park facilities primarily recycle renewable resources, thus generating large quantities of industrial waste, which renders potential risks from heavy metal contamination in the vicinity of the facilities^27^. This might also be an explanation for the enhanced joint synergistic effects of the main contaminant, As, with other heavy metals in this study.

However, this study highlights several limitations and areas for further study. First, relevant parameters on exposure were based on national standard values. When such information is directly employed at the local situation without sensitivity analysis, it may cause slight differences in health risk outcomes, although this was not a research focus of this study. Second, this study considered 1.0E-04 as the reference risk for *CR* and 1.0 as the reference risk for *HI*, based on practical experience in America and European countries^28^, because the applicability of corresponding Chinese references is still under discussion. This study also introduced the Geo-detector to recognize the main pollutants based on its functions, detecting the contribution of heavy metals to the soil contamination. However, when dealing with quantitative contaminants some prior knowledge, such as the impact of soil properties (e.g., pH values, soil humidity, soil type) on the mitigation-transformation mechanism of heavy metals, was essential for the discretization of these quantitative variables. Finally, although soil remediation is ongoing, integrating the environmental quality standards and human health risk assessment guidelines to supply scientific criteria is highly recommended as further work.

## Conclusions

To better identify contaminated areas and key contributing pollutants, this study proposed an integrated H-G scheme combining human health risk assessment model and Geo-detector methods based on the GIS technology; this study is the first application of this method. According to statistical analyses and spatial mapping results, areas contaminated with enough risk to require soil remediation were found in some residential regions rather than simply based on concentration. This result suggests that heavy metal contamination prevention strategies in this industrial park might be insufficient given the rapidly growing recycling industry of used and scrap electronic devices. Regarding human health effects, As and Cd are the main concerns due to their carcinogenic risks. These results confirm that the integrated H-G scheme proposed in this study can effectively identify risk areas polluted by heavy metals that really require soil remediation at a fine spatial resolution and can accurately target the contributing factors over a large geographical extent. The concentrations of critical As and Cd, as well as the associated exposed *CRs* are closed to safe thresholds after remediating the risk areas identified by the integrated H-G scheme. Therefore, the integrated H-G scheme provides an effective approach to support and guide decision-making for regional contaminated soil remediation.

## Materials and Methods

### Study area

**Figure 7:**
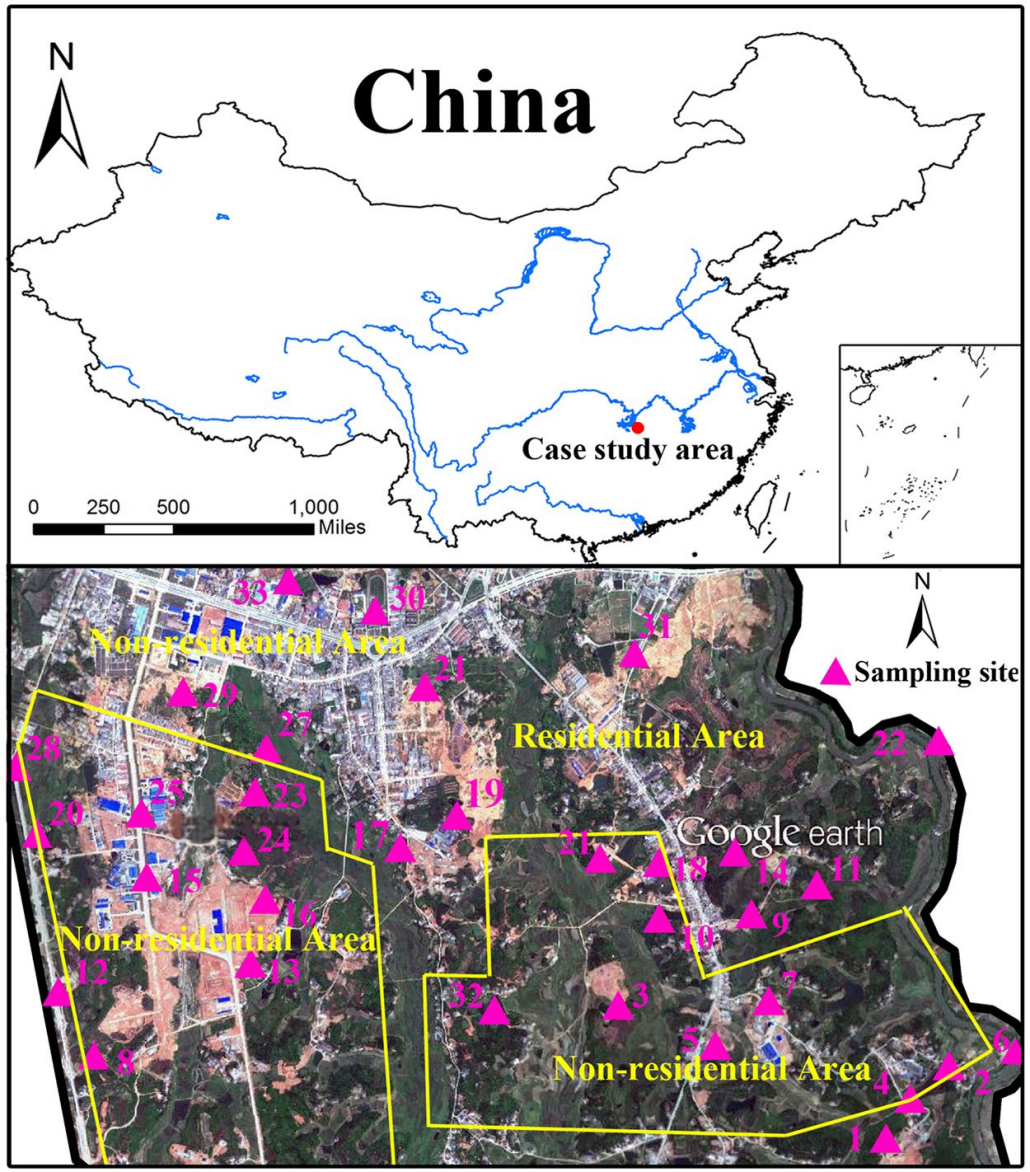
Study area and sampling locations (Note: Map data: Google earth, Digital Globe; ArcGIS 10.1 was used to create the map in this figure, http://www.esrichina.com.cn/2015/0107/2830.html).

The case study area, which is part of a resource recycling industrial park, is located in a city in southern China, covering 1.87 km^2^ (Fig. 7). With outmoded techniques and equipment, this industrial park produces large amounts of heavy metal waste, mainly cadmium (Cd), arsenic (As), copper (Cu), zinc (Zn) and mercury (Hg), from recycling electronic devices and refining oil from automobile tires. After becoming the first national pilot industrial park of a circular economy in 2005, heavy metal contamination in this area aggregated quickly, especially in the vicinity of working facilities. However, the residential usage in this area is still up to 52.9% because it is an area of craft production, which makes implementing fine-scale health risk assessment and soil remediation especially urgent in this area.

### Sampling and analytic method

For identifying the heavy metal polluted areas necessary for soil remediation, topsoil (at a depth of 0–20 cm) samples with average distance approximately 250 m at thirty-three sites were taken from the industrial park (Fig. 7), considering the distribution of recycling sources. To assess the reliability of the integrated H-G scheme, samples located at the places with heavy metals’ concentrations exceeding the grade II thresholds of soil environmental quality standard of China were recollected after remediation. Longitudes and latitudes of sampling locations were recorded by GPS receiver. Prior to measurement of heavy metal concentrations, soil samples were digested in a mixture of HF, HNO_3_ and HClO_4_. Then, we utilized atomic absorption spectrometry (China Standard GB/T 22105.1-2008) to analyze concentrations of Cd, Cu and Zn. Concentrations of As and Hg were measured by an atomic fluorescence spectrometer (China Standard GB/T 17138-1997 and GB/T 17141-1997). Quality assurance and quality control procedures were conducted by using standard reference material (GBW07401-GBW07408). All standard calibrations were prepared in the same acid matrix used for the soil samples. Meanwhile, this study performed the statistical analysis using IBM SPSS Statistics 19.0 for Windows.

### Human health risk assessment and geographical detector methods

The empirical methodology of this study is composed of three parts: IDW interpolation, Human health risk assessment, and Geo-detector analysis.

### Spatial distribution mapping by IDW interpolation

To recognize soil contamination from heavy metals across the entire industrial park more explicitly, this study applied the IDW spatial interpolation with ArcGIS (version 10.1) for mapping the spatial patterns of heavy metal concentrations. IDW is commonly used in spatial interpolation and has been introduced into contaminated site assessment^29^. It is a type of deterministic method for multivariate interpolation with a set of known scattered points. The values assigned to unknown points are calculated based on the weighted averages of values available at known points. It applies the inverse distance to each known point when assigning weights, given by

1 $$Z=\sum _{i=1}^{n}\frac{1}{{({D}_{i})}^{p}}{Z}_{i}/\sum _{i=1}^{n}\frac{1}{{({D}_{i})}^{p}}$$

where *Z* denotes the value of the interpolation points, *Z*_*i*_ (*i* = *1* ~ *n*) is the value of the sample points; *n* denotes the number of calculated sample points; *D*_*i*_ is the distance from sample point *i* to the interpolation point; and *p* is a positive power parameter determined by the minimum mean absolute error and significantly influences the outcome of interpolation. Additionally, ‘*n*-*1* cross validation’ was implemented to ensure the IDW interpolation accuracy in this study.

### Human health risk assessment for heavy metals

Human health risk assessment is a widely used to assess the potential health risk posed by heavy metals in soils to exposed people over a specified time period. The human health risk assessment model originating from the US EPA (USEPA 2007) has been recommended by the Environmental Protection Agency of China. According to the technical guidelines for risk assessment of contaminated sites in China (HJ/T 25-2014) and generally international environmental safety concerns^13^, the risks of heavy metals to local residents can be estimated using Eqs (2)–(5).

2 $$Risk=HI+CR=\sum _{i=1}^{m}\sum _{j=1}^{n}\frac{CDI}{RfD}+\sum _{i=1}^{m}\sum _{j=1}^{n}CDI\times SF$$

3 $$CD{I}_{oral}=\frac{{C}_{soil}\times IR\times ED\times EF\times CF}{BW\times AT}$$

4 $$CD{I}_{dermal}=\frac{{C}_{soil}\times ED\times EF\times CF\times SA\times AF\times ABS}{BW\times AT}$$

5 $$CD{I}_{particle}=\frac{{C}_{soil}\times PI\times DA\times ED\times EF\times fs\times CF}{BW\times AT}$$

where *HI* characterizes the total non-carcinogenic risk, and *CR* is the overall carcinogenic risk of all toxicants via exposure pathways, including oral ingestion, dermal contact and particle inhalation^17, 30^; *i* is one of three exposure routes, ingestion, dermal contact and particle inhalation; *j* represents the heavy metal contaminant; and *CDI* is the chemical daily intake of a contaminant for an individual (with 70-year as the life cycle), mg/(kg∙d); the relevant parameters of the model are listed in Table 4 (HJ/T 25-2014). *SF* for As and Cd is 1.50 (mg · kg^−1^ · d^−1^)^−1^ and 0.38 (mg · kg^−1^ · d^−1^)^−1^, respectively.

**Table 4:** Parameters employed for assessing human exposure risks.

Parameters	Meaning and value	Parameters	Meaning and value
*CDI*_*oral*_	*CDI* via ingestion, mg/(kg · d)	*CDI*_*dermal*_	*CDI* via dermal contact, mg/(kg · d)
*CDI*_*particle*_	*CDI* via particle inhalation, mg/(kg · d)	*AF*	skin adherence factor, 1, mg · cm^2^
*IR*	ingestion rate of soil, 100, mg/d	*BW*	body weight, 55.9, kg
*CF*	conversion factor, 10^−6^, kg · mg	*ED*	exposure duration, 30, a
*EF*	exposure frequency, 350, d/a	*SA*	surface area of the skin, 5000, cm^2^/d
*AT*	average time, 365 d · a^−1^ × 70, d	*C*_*soil*_	concentration of the exposure contaminant, mg/kg
*ABS*	absorption factor, 0.001	*PI*	retention fraction of inhaled particulates in body
*DA*	daily air inhalation rate, m^3^/d	*fs*	fraction of soil-borne particulates
*SF*	slope factor, kg · d/mg	*RfD*	reference dose, mg/(kg · d)

### Geo-detector analysis for predominant contaminants

As mentioned in the ‘Introduction’ section, based on spatial consistency of variables, Geo-detector was introduced to detect the main contaminant. In this study, the ‘Factor detector’ and ‘Interaction Detector’ were used. The calculation formula of its grounded *PD* is shown in Eq. (6).

6 $${\rm{PD}}=1-\frac{1}{N{\sigma }^{2}}\sum _{i=1}^{L}{N}_{i}{\sigma }_{i}^{2}$$

The whole area *N* designed to calculate *PD* is stratified into *L* strata, denoted by *i* = 1, …, *L* according to the concentration classification of heavy metals, defined as an attribute (the argument), whose statistical properties (e.g., mean and standard deviation) change over space. In Eq. (5), *N*_*i*_ and *σ* ^*2*^ denote the area and variance of the dependent variable, respectively, for each *i* stratum; *N* represents the whole area. *PD* ∈ [0, 1], *PD* = 1 means heavy metal concentration completely controls the overall risk, whereas *PD* = 0 means the concentration is completely unrelated to overall risk.

In this study, we first classified the overall health risk and heavy metal concentrations using a default interval classification and then loaded the distribution layers of all influential contaminants and the overall health risk into ArcGIS 10.1. After intersecting all layers, factor attributes of these layers were extracted and input into the Geo-detector model. The threshold for statistical significance was determined at *p* = 0.05. In this process, the overall health risk obtained by overlaying the carcinogenic and non-carcinogenic risk layers in ArcGIS 10.1 was employed as a dependent variable; each metal concentration in soil was taken as an independent variable to analyze the contribution of pollutants, Cd, As, Cu, Hg and Zn, to the total health risk level.
